# Fibrillarin-GFP Facilitates the Identification of Meiotic Competent Oocytes

**DOI:** 10.3389/fcell.2021.648331

**Published:** 2021-04-15

**Authors:** Ting Wang, Jie Na

**Affiliations:** ^1^Center for Stem Cell Biology and Regenerative Medicine, School of Medicine, Tsinghua University, Beijing, China; ^2^Tsinghua-Peking Center for Life Sciences, Tsinghua University, Beijing, China

**Keywords:** nucleolus, fibrillarin, meiosis, oocyte maturation, transcriptome analysis

## Abstract

The nucleolus undergoes significant functional changes and plays important roles during mammalian oocyte meiotic maturation. Fibrillarin (FBL) is the component of nucleolar small nuclear ribonucleoprotein (snRNP) particle and localizes to the dense fibrillar component (DFC) of the nucleolus. We found that FBL–GFP displays an uneven and cloudy localization in the nucleolus of non-surrounded nucleolus (NSN) oocytes, while it distributes evenly and to a few bright dots in the surrounded nucleolus (SN) oocytes. Accordingly, NSN oocytes showed active nascent RNA transcription, while the SN group was transcriptionally quiescent. NSN geminal vesicles also contained more DNA damage marker γH2AX foci. Based on different FBL–GFP patterns in live oocytes, the ones with superior meiotic maturation potential can be identified. Global transcriptome profiling revealed a significant difference in single SN and NSN oocytes. Thus, FBL–GFP can serve as a marker for nucleolus activity, which also correlates with transcription activity and the quality of oocytes.

## Introduction

The nucleolus is important for mammalian oocyte meiotic maturation. For all mammalian species, oocytes are arrested in the prophase with a large nucleus called the germinal vesicle (GV). During oocyte meiosis I (M I), the germinal vesicle breakdown (GVBD), chromosomes condense and align at the metaphase I spindle. Then, the homologous chromosomes separate during anaphase I and the oocytes protrude the 1st polar body. After completing the first meiotic division, oocytes are arrested at metaphase II, waiting to be fertilized (Fulka et al., 1998). The nucleolus of mammalian oocytes is termed nucleolus-like body (NLB) due to their unusual ultrastructure and different functions from the active somatic nucleolus. During oogenesis, transcriptionally active nucleoli in growing oocytes transform to NLBs in fully grown oocytes. After fertilization and zygotic genome activation, NLBs return to active nucleoli in preimplantation embryos. NLBs are composed of densely packed homogenous fibrillar material. By contrast, somatic fibrillo-granular nucleoli are assembled around nucleolar organizer regions (NORs) (Boisvert et al., 2007), which are divided into subregions named fibrillar centers (FC), dense fibrillar components (DFC), and granular components (GC) (Carmo-Fonseca et al., 2000). Fibrillarin (FBL) is an snRNP member that can form protein clusters in DFC and is involved in 47S pre-rRNA processing (Boisvert et al., 2007; Yao et al., 2019). Meanwhile, nucleolar rRNA transcription and processing status correlated with nuclear chromatin configuration.

In the mouse, it has been reported that fully grown oocytes show heterogeneous chromatin configuration (Zuccotti et al., 1995). Those with a ring of fully condensed chromatin surrounding the NLB are termed surrounded nucleolus (SN), while the others with less condensed chromatin rim surrounding the NLB are called non-surrounded nucleolus (NSN). More than 90% of oocytes in 2-week-old female mice are NSN oocytes arrested in the prophase of meiosis I. As the oocytes grow, some stay in the NSN configuration, whereas others undergo an NSN-to-SN transition (Zuccotti et al., 1995). As the chromatin configuration of the SN and NSN nucleus differs considerably, so does their transcription activity. NSN oocytes are transcriptionally active, where RNA Pol I and RNA Pol II transcribe rRNA and mRNA, respectively. On the other hand, SN oocytes are transcriptionally inactive (Bouniol-Baly et al., 1999).

SN oocytes have been shown to reach metaphase II at a high frequency during *in vitro* maturation culture, while NSN oocytes have limited meiosis competence and developmental potential (Ogushi et al., 2008; Fulka and Aoki, 2016). After fertilization, most SN oocytes can develop to blastocyst stage. On the contrary, fertilized NSN oocytes are often arrested at the two-cell stage (Zuccotti et al., 2002). The above studies indicated that the conformation of NLB in GV oocytes could be a marker of meiotic competence and developmental potential.

In this work, we visualize the NLB in live GV oocytes using an FBL–GFP fusion protein. We show that fully grown oocytes could be classified into two NSN and SN types based on the FBL–GFP distribution pattern. Besides, we analyzed the transcriptome heterogeneity of single NSN and SN GV oocytes and MII oocytes derived from them. Our study provided new tools and resources to evaluate the quality of mammalian oocytes.

## Materials and Methods

### Oocyte Collection and Culture

All animal experiments were conducted following the Guide for the Care and Use of Animals for Research Purposes. The protocol for mouse oocyte isolation was approved by the Institutional Animal Care and Use Committee and Internal Review Board of Tsinghua University. GV oocytes were collected from the ovary of 3- to 4-week-old C57BL/6 females (Charles River) 48 h post-PMSG (San-Sheng Pharmaceutical Co., Ltd.) in M2 medium (Sigma M7167) supplemented with 10 μM Cilostamide (Cayman 14455), then cultured in IVM medium at 37.5°C in a CO2 incubator. An IVM medium (Hayashi and Saitou, 2013) was freshly prepared by mixing α-MEM (Gibco C12571) and 10% FBS (BI 04-001-1ACS), supplemented with 0.025 mg/ml sodium pyruvate (Sigma P4562) and 0.08 mg/ml gentamicin (Invitrogen 15750-060), with or without 10 μM Cilostamide (Cayman 14455).

### Plasmid Construction, *in vitro* mRNA Synthesis, and Microinjection

Mouse fibrillarin was subcloned into the RN3P vector for *in vitro* transcription of mRNA. Capped mRNAs were generated using a T3 mMESSAGE mMACHINE Kit (Thermo Fisher Scientific, AM1348) following the manufacturer’s instructions. Microinjection of mRNA at desired concentrations into mouse GV oocytes was performed on a Leica DMI3000B microscope equipped with a Leica micromanipulator as previously described (Na and Zernicka-Goetz, 2006).

### Immunofluorescence Staining and EU Staining

Mouse oocytes were first treated with acidic Tyrode’s solution (Merck Millipore MR-004-D) to remove the zona pellucida, then fixed with either 4% PFA in PBS for 20 min at room temperature or ice-cold 100% methanol for 20 min at –20°C. After fixation, oocytes were permeabilized with 0.5% Triton X-100 for 20 min and blocked with 3% BSA in PBST (0.1% Tween in PBS) for 2 h at room temperature. Primary antibody incubation was carried out at 4°C overnight. The primary antibodies include FBL (ABclonal A1136) and anti-gamma H2A.X (Abcam ab26350). The samples were then washed in PBST and incubated with secondary antibodies, including Dylight 488 Goat Anti-Rabbit (EarthOx E032220) and Dylight 549 Goat Anti-Mouse (EarthOx E032310) at 4°C overnight. The nucleus was stained with DAPI (Sigma D9542) or Hoechst 33342 (Dojindo H342). After staining, oocytes were mounted on coverslips in VECTASHIELD mounting medium (Vectorlabs H-1000). For EU incorporation assay, EU was injected into the oocyte cytoplasm at a concentration of 100 mM. Newly synthesized RNA was detected following the manufacturer’s instruction (RiboBio C10316).

### Enzymatic Antigen Retrieval

For FBL immunostaining, we performed enzymatic antigen retrieval to retrieve the fibrillarin epitope first. Oocytes were fixed as described above and then treated with 1 μg/ml proteinase K (Transgen GE201-01) for 20 min. After three washes in PBST, oocytes were postfixed with 4% PFA in PBS for 20 min and treated with 0.2% Triton X-100 for 10 min, followed by conventional immunofluorescence staining.

### Imaging and Quantifications

Images were acquired on a Nikon A1R HD25 confocal microscope. Images were processed using Fiji software (Schindelin et al., 2012). For fluorescence intensity distribution analysis, a line across the oocyte diameter was drawn, and fluorescence levels were determined using Plot Profile function. Foci numbers were calculated using 3D Objects Counter function.

### High-Throughput RNA-Sequencing Library Construction

For the oocyte gene expression study, we used the SMART-seq2 protocol to amplify single oocyte RNA (Picelli et al., 2014). The single oocyte was first lysed in hypotonic lysis buffer (Amresco, M334), followed by reverse transcription and pre-amplification of cDNA. After AMPure XP beads (Beckman Coulter A63881) purification, cDNAs were tagmented by Tn5 to obtain Illumina Nextera libraries. All libraries were sequenced on Illumina HiSeq X-10 according to the manufacturer’s instruction.

### RNA-Sequencing Data Analysis

Adapter sequences were trimmed using TrimGalore (v0.4.4) (Krueger, 2017). Clean reads were mapped to the mouse genome (mm10) using Bowtie2 (v2.3.5) (Langmead and Salzberg, 2012) software with the Ensembl Annotation. Read counts and Fragments Per Kilobase per Million mapped reads (FPKM) of Refseq genes were calculated by RSEM (v1.2.28) (Li and Dewey, 2011). The normalization of read counts was completed by DESeq2 package (v1.20.0) (Love et al., 2014). Differentially expressed genes (DEGs) were selected according to a previous report (Ma et al., 2013). Then genes with a threshold of absolute fold change ≥ 1.5 were regarded as DEGs. Principle component analysis was performed using R’s “prcomp” function and drawn by ggplot2 package (v3.1.0) (Ginestet, 2011). The heatmaps were produced by the heatmap2 function of the gplots package (v3.0.1.1) (Warnes et al., 2015) with the hierarchical clustering method. The soft clustering of gene expression data is implemented using the fuzzy c-means algorithm by the Mfuzz package (v2.42.0) (Futschik and Carlisle, 2005). The enrichment of GO was analyzed using the clusterProfiler package (v3.8.1) (Yu et al., 2012).

### Statistical Analysis

Data were presented as means ± standard error of the mean (s.e.m.). Statistical significance was determined by unpaired two-tailed Student’s *t*-test using GraphPad Prism software. *P* < 0.05 was considered significant.

## Results

### FBL–GFP Marks Nucleolus in Live SN and NSN GV Oocytes

**FIGURE 1:**
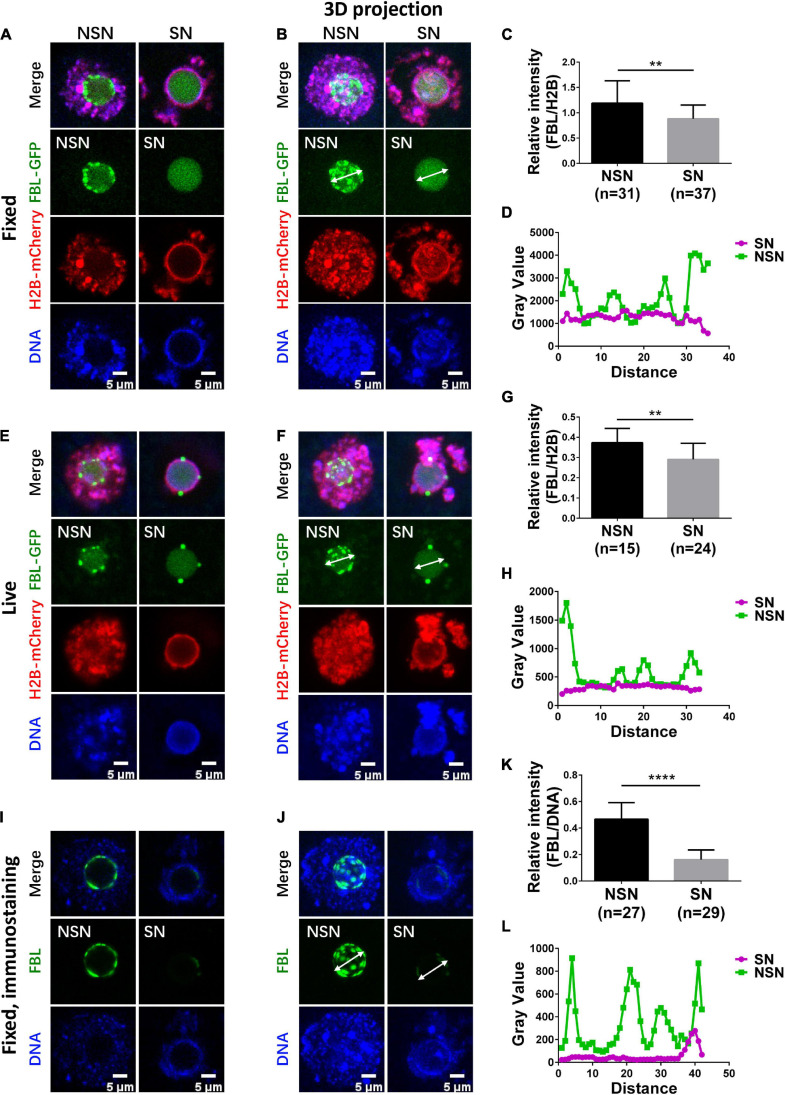
Organization of FBL clusters in NSN and SN oocytes. **(A,B)** FBL exhibits a clustered distribution pattern in fixed NSN GV oocytes. The clusters decrease significantly in fixed SN GV oocytes. Representative z section **(A)** and 3D projection **(B)** confocal images of exogenously expressed FBL–GFP and H2B-mCherry in oocytes are shown. Scale bar = 5 μm. **(C)** Bar graph shows relative FBL signal intensity to H2B signal intensity in fixed NSN and SN GV oocytes. *N* = 68. **P < 0.01. **(D)** The intensity distribution of FBL was measured along the arrow lines drawn on the images in **(B)**. Green, NSN; purple, SN. **(E,F)** FBL distribution patterns in live NSN and SN GV oocytes. Representative z section **(E)** and 3D projection **(F)** confocal images of exogenously expressed FBL–GFP and H2B-mCherry in oocytes are shown. Scale bar = 5 μm. **(G)** The bar graph shows relative FBL signal intensity to H2B signal intensity in live NSN and SN GV oocytes. *N* = 39. **P < 0.01. **(H)** The intensity distribution of FBL was measured along the arrow lines drawn on the images in **(F)**. Green, NSN; purple, SN. **(I,J)** FBL distribution patterns were confirmed with anti-FBL in fixed NSN and SN oocytes. Representative z section **(I)** and 3D projection **(J)** confocal images of endogenous FBL localization are shown. Scale bar = 5 μm. **(K)** The bar graph shows relative anti-FBL signal intensity to DNA signal intensity in fixed NSN and SN GV oocytes. *N* = 56. ****P < 0.0001. **(L)** The intensity distribution of anti-FBL was measured along the arrow lines drawn on the images in **(J)**. Green, NSN; purple, SN.

To visualize the NLB in GVs, we expressed GFP-tagged FBL by microinjection of FBL–GFP mRNA into the cytoplasm of GV oocytes; H2B-mCherry mRNA was co-injected. Oocytes were blocked in the GV stage with Cilostamide. We first used confocal microscopy to examine fixed GV oocytes. FBL–GFP formed numerous bright aggregates of various sizes. These aggregates appeared to locate on the surface of NLB. FBL–GFP also has a cloudy distribution throughout the NLB. DAPI staining indicates that GV oocytes with such FBL–GFP localization are NSN GV oocytes (Figure 1A), which is in agreement with earlier reports (Zatsepina et al., 2000). By contrast, in SN GV oocytes, few bright FBL–GFP aggregates can be observed on the NLB surface (Figure 1A). After the maximum projection of all stacks, NSN GV oocytes exhibited a lumpy FBL distribution pattern compared to SN GV oocytes (Figure 1B). We normalized FBL–GFP levels against co-injected H2B-mCherry. The fluorescence quantification showed that the amount of FBL–GFP protein is significantly less in SN oocytes than in NSN oocytes (Figure 1C). We also measured the gray value across the NLB. In NSN GV oocytes, the gray value of FBL–GFP fluorescence had multiple high peaks along the line drawn across one typical NLB (Figure 1D). In SN GV oocytes, the FBL–GFP gray value was generally lower across nucleolus, with only one or two peaks (Figure 1D). Thus, based on the above results, NSN GV oocytes could be easily distinguished from SN GV oocytes.

Next, we imaged live NSN and SN GV oocytes; FBL–GFP displayed similar fluorescence levels and localization patterns in the NLB as in fixed oocytes (Figures 1E–H). Finally, we performed immunostaining of endogenous Fibrillarin using an FBL antibody. As expected, endogenous FBL showed similar differences in NSN and SN GV oocytes (Figures 1I–L), and the protein level of endogenous Fibrillarin in NSN GV oocytes was more than twice that in SN oocytes (Figure 1K). Taken together, FBL–GFP can be used as a convenient tool to distinguish live SN GV oocytes from NSN ones in real time.

### Nucleolus Activity Predicts GV Oocyte Meiotic Maturation Potential

**FIGURE 2:**
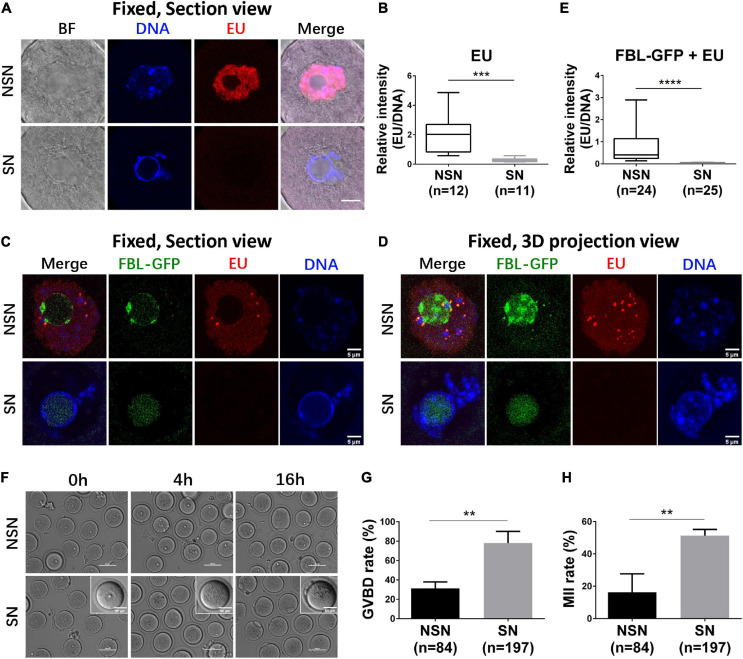
The transcription activity of NSN and SN GV oocytes. **(A,B)** Representative z section images of nascent RNA transcription in NSN and SN GV oocytes. Box–whisker plot shows relative EU signal intensity to DNA signal intensity in NSN and SN GV oocytes. Scale bar = 10 μm. *N* = 23. ***P < 0.001. **(C–E)** Representative z section **(C)** and 3D projection **(D)** confocal images of nascent RNA transcription in FBL–GFP-classified NSN and SN GV oocytes. Box–whisker plot shows relative EU signal intensity to DNA signal intensity in FBL–GFP-classified NSN and SN GV oocytes. Scale bar = 5 μm. *N* = 49. ****P < 0.0001. **(F–H)** Representative live oocyte bright-field images at 0, 4, and 16 h after FBL–GFP classification and Cilostamide release. Scale bars = 100 or 50 μm. The GVBD rate at 4 h was shown in **(G)**. The 1st polar body extrusion rate at 16 h was shown in **(H)**. **P < 0.01.

The full-grown meiotic competent GV oocytes are mostly SN oocytes and transcriptionally quiescent (Fulka and Aoki, 2016). To test whether the FBL–GFP pattern correlates with different transcriptional activities, we performed 5-ethynyl uridine (EU) incorporation assay. In the first series of experiments, we used Hoechst 33342 labeling to distinguish NSN and SN GV oocytes. GV oocytes with diffused Hoechst 33342 nucleus distribution have apparent positive EU staining, indicating that they are NSN oocytes that transcribe new RNAs. On the other hand, GV oocytes with a prominent ring-like DNA staining have a minimal EU signal, suggesting that they are transcriptionally quiescent (Figure 2A). Quantification of EU fluorescence intensity confirmed the observation; NSN oocytes have significantly more EU incorporation than SN oocytes (Figure 2B). Then, we microinjected mRNA encoding FBL–GFP and separated NSN and SN GV oocytes based on FBL–GFP localization. The EU signal was much stronger in oocytes with NSN type of FBL–GFP distribution, while GV oocytes with smooth FBL–GFP distribution virtually had no EU signal, indicating that they are SN oocytes (Figures 2C,D). Since FBL played important functions in pre-rRNA processing (Boisvert et al., 2007; Yao et al., 2019), the observations that SN oocytes showed substantially reduced FBL levels (Figures 1C,G,K) and an almost undetectable EU signal (Figure 2E) are consistent with previous knowledge that full-grown GV oocytes are transcriptionally quiescent before meiosis.

Next, we compared the *in vitro* maturation ability of SN and NSN GV oocytes classified based on FBL–GFP distribution (Figure 2F). NSN oocytes showed a significantly lower GVBD rate (Figure 2G) and 1st polar body extrusion rate (Figure 2H) than SN oocytes. The above results demonstrated that FBL–GFP could be used to select SN and NSN oocytes for meiotic maturation evaluation.

### Gene Expression Revealed the Difference Between NSN and SN Oocytes Before and After Meiosis I

**FIGURE 3:**
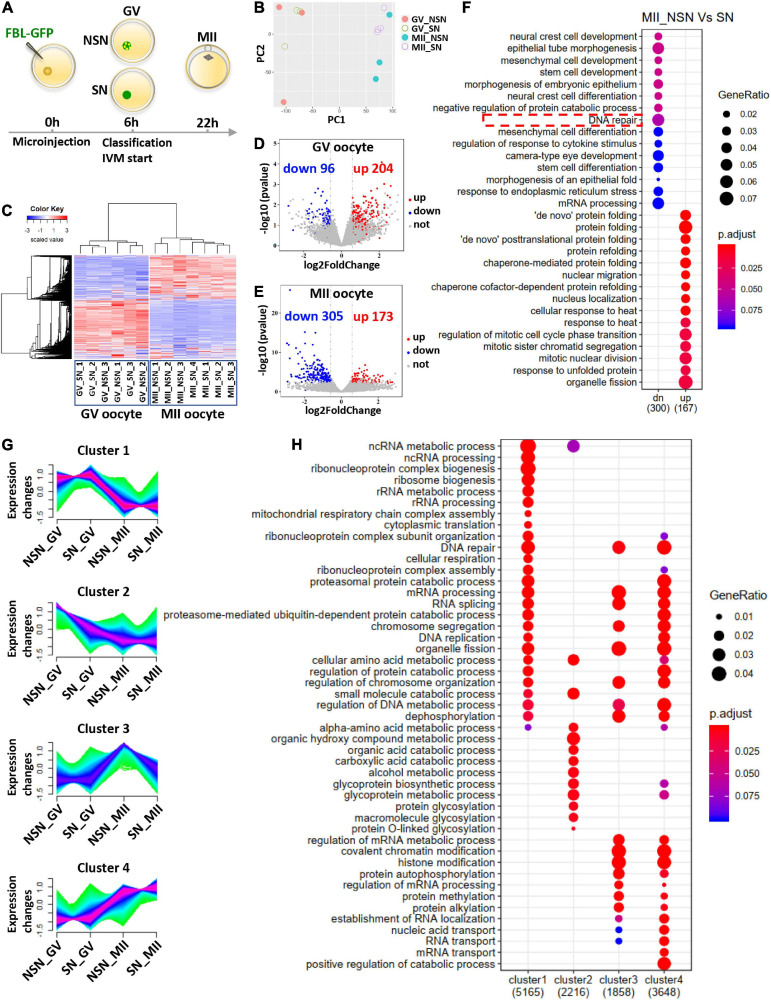
Transcriptome comparison before and after meiotic maturation of NSN and SN oocytes. **(A)** Schematic illustrating the pipeline of preparing single oocyte RNA-Seq samples. **(B)** PCA analysis of RNA-Seq data from NSN and SN oocytes at the GV and MII stages. Each dot represents one library, color-coded by oocyte type and stage. **(C)** Gene expression heatmap of NSN and SN oocytes at the GV stage and MII stage. The blue to red color change represents the transcription level from low to high. **(D,E)** Volcano plot of DEGs in NSN oocytes vs. SN oocytes at the GV stage and MII stage. The upregulated (fold change >1.5) and downregulated (fold change <–1.5) transcripts in each comparison are labeled by red and blue, respectively (*P* < 0.05). **(F)** Gene ontology analysis of the upregulated and downregulated DEGs between NSN and SN MII oocytes. **(G)** Mfuzz analysis of RNA-Seq data from NSN and SN oocytes at the GV and MII stages. The x axis represents oocyte types and stages, while the y axis represents standardized FPKM. Green to purple colored lines correspond to genes with low to high membership value. **(H)** Gene ontology analysis of genes in the four clusters from **(G)** (see also Supplementary Tables 1–3).

To characterize the transcriptome difference during NSN and SN oocyte maturation, we carried out single oocyte RNA-seq of NSN and SN GV oocytes; MII oocytes matured from NSN and SN GV oocytes (Figure 3A). Then, we performed principal component analysis (PCA) and hierarchical cluster analysis. The PCA graph showed that the transcriptome of single GV and MII oocytes separated into two groups (Figure 3B). Although the transcriptome of single NSN or SN GV oocytes sometimes clustered together, GV stage and MII stage oocytes have very different gene expression patterns (Figures 3B,C). The single oocyte analysis revealed heterogeneity of the NSN and SN oocyte transcriptome and suggested that despite that NSN GV oocytes are mostly transcriptionally active, their overall transcriptomes are still similar to SN GV oocytes. We compared differentially expressed genes (DEGs) in NSN and SN oocytes at GV or MII stages, respectively (Figures 3D,E). 204 upregulated genes and 96 downregulated genes were identified in NSN vs. SN oocytes at the GV stage (Figure 3D and Supplementary Table 1). In the MII pairwise comparison, 173 genes were upregulated in NSN oocytes, and 305 were downregulated (Figure 3E), indicating that after meiotic maturation, MII oocytes derived from NSN and SN GV oocytes became more distinct from each other. Gene ontology (GO) analysis of MII oocyte DEGs highlighted pathways involving DNA repair, nuclear migration, and nucleus localization (Figure 3F and Supplementary Table 2).

We also performed a more detailed analysis about the change in gene expression pattern, taking into account both NSN and SN oocytes and their meiotic stages. Four gene clusters with distinct temporal expression kinetics were classified (Figure 3G). Among these, cluster 1 contained genes most highly expressed in SN GV oocytes and downregulated in MII oocytes, while cluster 2 was comprised of genes with the highest expression in NSN GV oocytes, then downregulated in MII. Cluster 1 and cluster 2 were enriched for very different biological processes. For example, various ncRNA-, ribosomal RNA-, and mRNA-processing genes were enriched in cluster 1, suggesting that the RNA processing and metabolism might be more complete in SN GV oocytes, while genes related to the organic acid catabolic process and protein glycosylation were more abundant in NSN GV oocytes, implying that NSN oocytes might not be metabolically as mature as SN GV oocytes (Figure 3H). Cluster 3 genes were the most upregulated in MII oocytes derived from NSN GV oocytes (NSN-MII). It contained more mRNA-processing genes and protein methylation, alkylation genes, indicating that certain aspects of the mRNA and protein processing were incomplete in this oocyte group. Cluster 4 genes were significantly higher in the MII stage compared to the GV stage. Besides, the levels of this group of genes were the highest in MII derived from SN GV oocytes (SN-MII). Genes highly represented in cluster 4 include proteasome-mediated ubiquitin-dependent protein catabolic processes, RNA localization, and transport, which is in accordance with the metaphase II arrest status of the oocytes. Interestingly, more DNA repair genes were in cluster 4 than cluster 3, suggesting that SN-MII oocytes might be more capable of repairing DNA damage and handling genomic stress than NSN-MII oocytes (Figure 3H). The enriched biological process in each cluster and genes in each biological process were provided in Supplementary Table 3.

**FIGURE 4:**
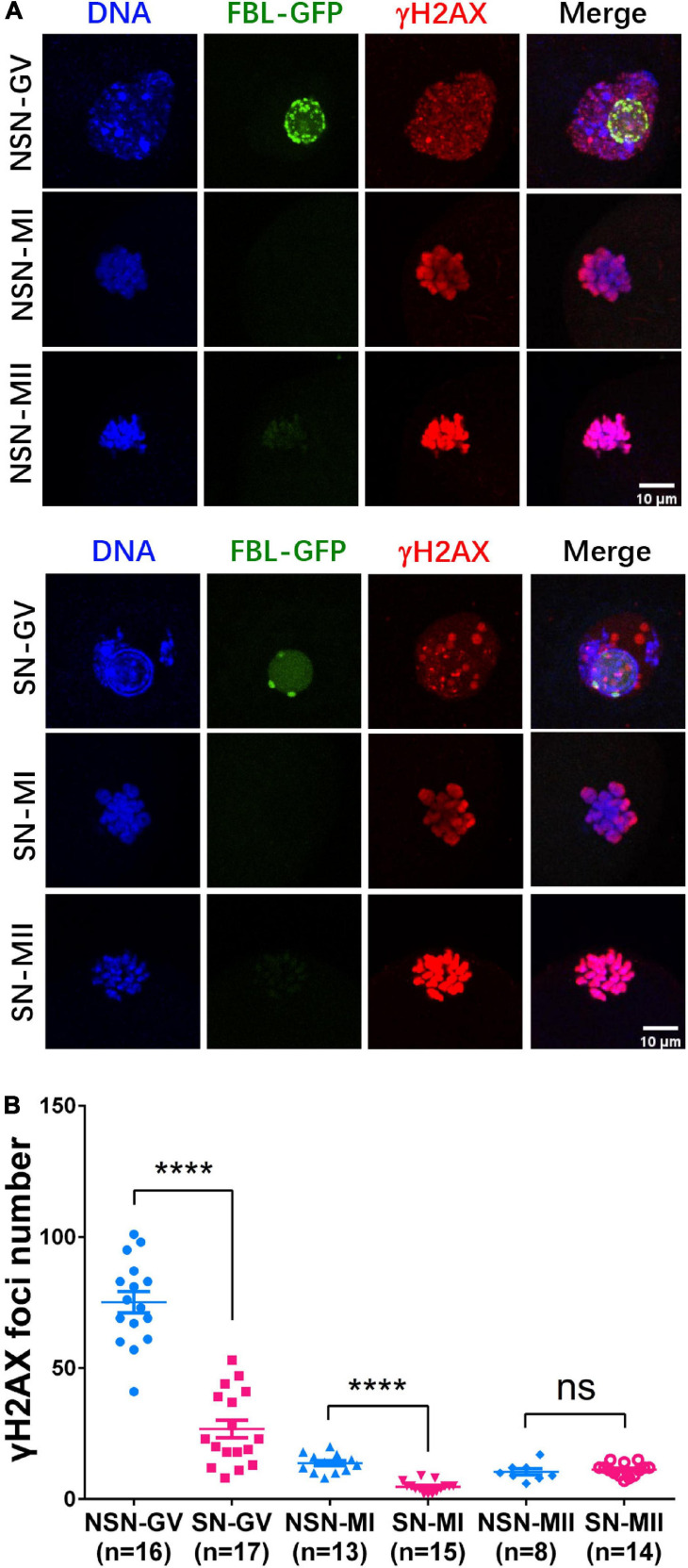
H2AX phosphorylation dynamics during meiotic maturation. **(A)** γH2AX staining in NSN and SN oocytes at GV, MI, and MII stage. Scale bars = 10 μm. **(B)** The scatter plot shows the quantification of γH2AX foci numbers in **(A)**. ****P < 0.0001.

Since DNA repair genes were downregulated in NSN-MII oocytes, we next investigated H2AX phosphorylation during meiotic maturation by immunostaining. The background of γH2AX in the nucleus was higher, and the number of γH2AX foci was significantly more in NSN GV oocytes than SN oocytes (Figure 4A). The γH2AX foci number in NSN oocytes was about 2.8 times that in SN oocytes at both GV and MI stages (Figure 4B), indicating that NSN oocytes are more susceptible to DNA damage and may explain their inferior meiotic competence.

## Discussion

In this study, we showed that FBL–GFP localizes nicely to NLB in mouse GV oocytes and could be a reliable new marker to distinguish NSN and SN status in live oocytes. It allowed direct visualization of nucleolus activity in real-time live oocytes. The large and round nucleolus in GV oocytes is quite distinct from the reticulated nucleolus in somatic cells and ESCs (Kyogoku et al., 2014). The FBL–GFP nicely demarcated the nucleolus in NSN and SN GV oocytes, and its pattern was very similar to the immunostaining of endogenous FBL in our study and others (Zatsepina et al., 2000). The numerous FBL–GFP dots in NSN GV reflected that both Pol I and Pol II were still transcribing. There was substantial EU staining in the nucleus but not inside the nucleolus, suggesting that the newly transcribed rRNA was immediately exported into the nucleoplasm as described in Yao et al.’s (2019) study. Interestingly, the nucleolus from GV oocytes, but not zygotes, somatic cells, or embryonic stem cells (ESCs), was required for the development of naturally fertilized eggs and somatic nucleus transferred embryos (Ogushi et al., 2008). Removing the nucleolus from GV oocytes leads to abnormal chromatin remodeling, reduced expression of the centric and pericentric satellite DNA in zygotes, and development failure (Fulka and Langerova, 2014; Kyogoku et al., 2014). Our result and several studies also showed that SN GV oocytes have much better meiotic maturation potential than NSN GVs. Thus, it would be of future interest to examine the relationship of NSN, SN nucleolus, and the surrounding heterochromatin using FBL–GFP and centromere markers in live oocytes.

In live NSN and SN GV oocytes, the FBL–GFP pattern in the nucleolus co-related well with the H2B-mCherry distribution outside the nucleolus. After selecting NSN and SN GV oocytes based on FBL–GFP, they can resume meiosis I and be *in vitro* fertilized to study their developmental potential. Using live-cell DNA dye Hoechst to stain DNA in the GV can have variable results and may cause DNA damage to the oocytes if subsequent meiosis, fertilization, and embryo development are desired. Judging by the space between zona and the oocyte membrane is convenient (Inoue et al., 2008) but is not always consistent with the nucleolus activity, especially when GV oocytes are arrested in the GV stage during *in vitro* culture. Our transcriptome study also revealed differential gene expression associated with NSN and SN GV oocyte characteristics and MII oocytes derived from them. Notably, the number and levels of DNA repair and developmental genes were reduced in NSN MII oocytes, while several metabolic pathway genes and epigenetic regulation genes were different in NSN and SN GVs. Indeed, immunostaining of γH2AX showed that there were significantly more γH2AX foci in NSN GV and MI oocytes. It is possible that NSN oocytes were less able to maintain the mRNA levels of genes essential for the repair of DNA damage or other developmentally critical processes due to their immature metabolic and epigenetic status and RNA processing capacity, which eventually resulted in their poor developmental potential.

Our approach also has some limitations. For example, cytoplasmic microinjection of mRNA may cause additional damage to GV oocytes. Nevertheless, our results about FBL–GFP offered a useful new tool for observing nucleolus activity in live GV oocytes.

In summary, our study showed that FBL–GFP could be a useful tool for indicating nucleolus activity and separating live NSN and SN GV oocytes. The transcriptome profiles of single NSN and SN GV oocytes, and their derivative MII oocytes, provided a valuable resource that might guide the quality control of mammalian oocyte meiotic maturation.

## Data Availability Statement

The datasets generated for this study can be found in the online repositories. The names of the repository/repositories and accession number(s) can be found below: https://www.ncbi.nlm.nih.gov/geo/, GSE163935.

## Ethics Statement

The animal study was reviewed and approved by the Institutional Animal Care and Use Committee and Internal Review Board of Tsinghua University.

## Author Contributions

TW and JN conceived the study, designed the experiments, and wrote the manuscript. TW performed the mouse oocyte microinjection, immunostaining, confocal microscopy, RNA-seq library construction, and data analysis. Both authors contributed to the article and approved the submitted version.

## Conflict of Interest

The authors declare that the research was conducted in the absence of any commercial or financial relationships that could be construed as a potential conflict of interest.
