# Supplementary material for: Fibrillarin-GFP Facilitates the Identification of Meiotic Competent Oocytes
Source: Front Cell Dev Biol. 2021 Apr 15;9:648331. doi: 10.3389/fcell.2021.648331 (PMC8082495; doi:10.3389/fcell.2021.648331)
Supplement: Supplementary file 1 [file Data_Sheet_1.PDF]

Supplementary Material

1 Supplementary Tables

Table S1. Differentially expressed genes and enriched GO terms in NSN and SN GV oocytes. (related to Figure 3D)

Table S2. Differentially expressed genes and enriched GO terms in NSN and SN MII oocytes. (related to Figure 3E)

Table S3. Enriched GO terms of the 4 Mfuzz clusters with distinct temporal expression kinetics. (related to Figure 3H)
